# Supplementary material for: A Gas Sensors Detection System for Real-Time Monitoring of Changes in Volatile Organic Compounds during Oolong Tea Processing
Source: Foods. 2024 May 30;13(11):1721. doi: 10.3390/foods13111721 (PMC11171579; doi:10.3390/foods13111721)
Supplement: Supplementary file 1 [file foods-13-01721-s001.zip › foods-3014687-supplementary.pdf]

A Gas Sensors Detection System for Real-Time Monitoring of Changes in Volatile Organic Compounds during Oolong Tea Processing

Zhang Han [1], Waqas Ahmad [2], Yanna Rong [2], Xuanyu Chen [2], Songguang Zhao [2], Jinghao Yu [2], Pengfei Zheng [2,3], Chunchi Huang [3] and Huanhuan Li [2,*]

[1] School of Mechanical Engineering, Jiangsu University, Zhenjiang 212013, China; hello1hz@163.com
[2] School of Food and Biological Engineering, Jiangsu University, Zhenjiang 212013, China; waqas3ahmad@gmail.com (W.A.); ryn981020@163.com (Y.R.); swchenxuanyu@gmail.com (X.C.); zsgemail9679@163.com (S.Z.); yujinghao878@163.com (J.Y.); 17853260667@163.com (P.Z.)
[3] Chichun Machinery (Xiamen) Co., Ltd., Xiamen 361100, China; huangchunchi@126.com
* Correspondence: jiona1044@ujs.edu.cn; Tel.: +86-511-88790318; Fax: +86-511-88780201

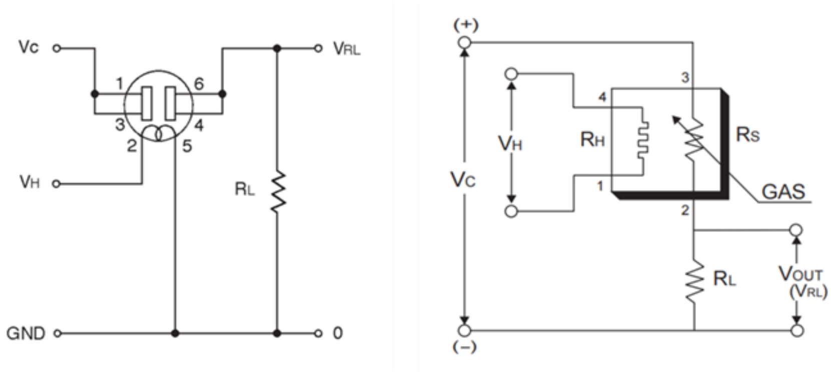

Figure S1. For circuit schematics of sensors of both structures, refer to the product manual of Figaro GMBH.

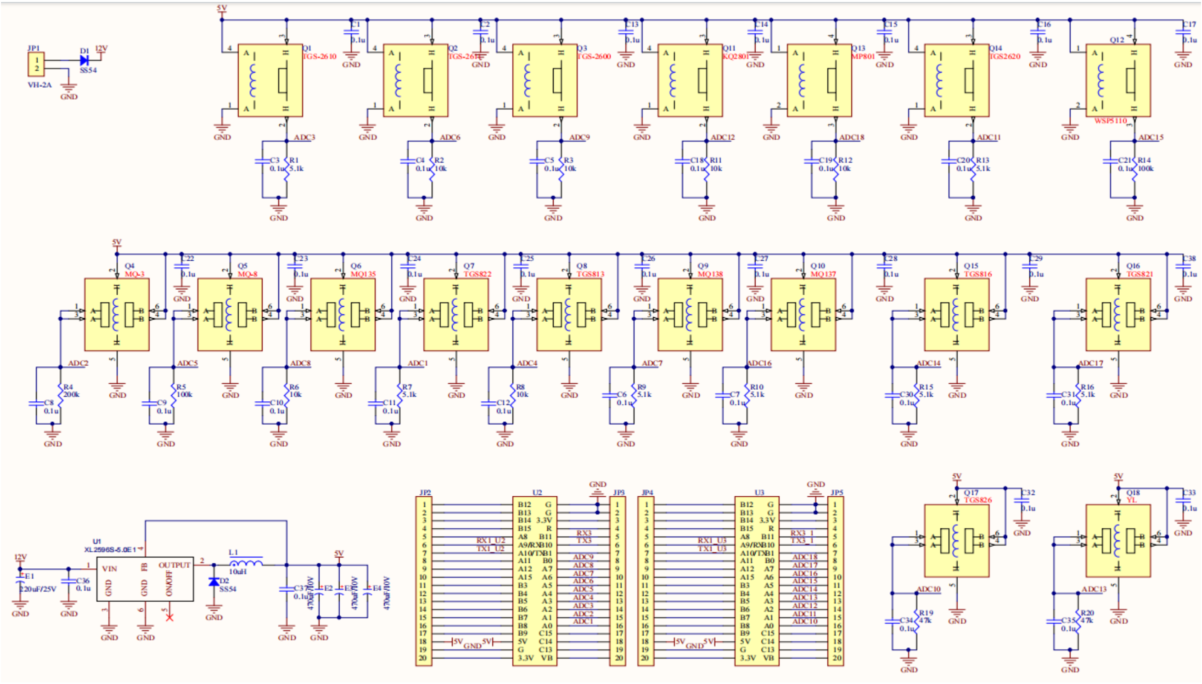

Figure S2. Gas sensors detection system circuit integrated schematics used in the current work.

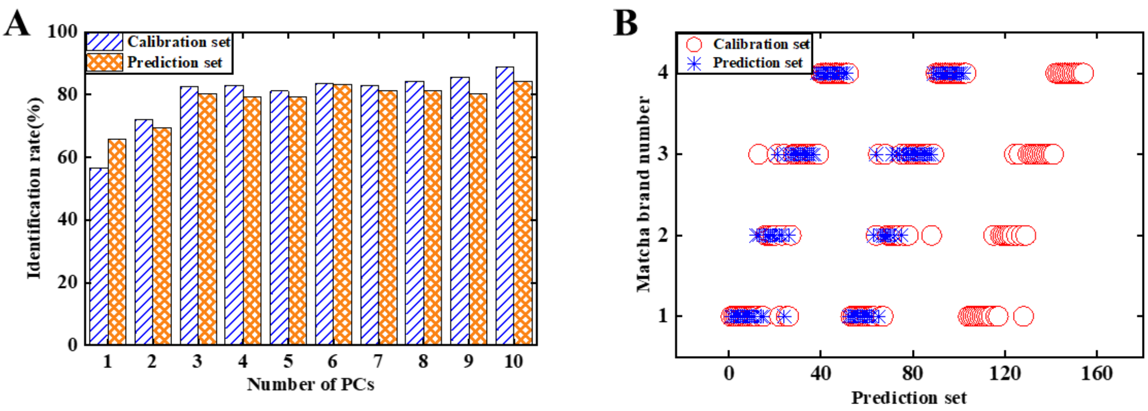

Figure S3. The results of LDA models.

Table S1. Eigenvalue and cumulate contribution rates of the principal components.

Principal Component Number	Eigenvalue	Percentage of Variance (%)	Cumulative (%)
1	6.26008	48.1545	48.1545
2	4.68756	36.05816	84.21266
3	1.53785	11.82963	96.04229
4	0.31198	2.39985	98.44213
5	0.09539	0.73377	99.17591
6	0.05303	0.4079	99.5838
7	0.02258	0.17367	99.75747
8	0.01434	0.11032	99.8678
9	0.00689	0.05298	99.92078
10	0.00538	0.04138	99.96215

Table S2. Cross matrix for the identification results of models in the calibration and prediction set.

Models	Grade	NC [a]	Results in the calibration set					NP [b]	Results in the prediction set				
			H	F1	F2	F3	CR [c]		H	F1	F2	F3	CR [c]
LDA	H	38	34	10	0	0	83.77%	26	22	4	0	0	83.33%
	F1	39	2	22	3	0		25	3	16	0	0	
	F2	38	2	7	34	0		26	1	5	23	0	
	F3	39	0	0	1	39		25	0	0	3	25	
KNN	H	38	36	3	0	0	93.51%	26	24	1	0	0	88.24%
	F1	39	0	33	1	0		25	2	20	3	0	
	F2	38	2	3	36	0		26	0	4	21	0	
	F3	39	0	0	1	39		25	0	0	2	25	
LeNet5	H	38	34	2	0	0	92.86%	26	23	1	0	0	89.21%
	F1	39	2	33	1	0		25	3	20	0	0	
	F2	38	2	4	37	0		26	0	4	24	1	
	F3	39	0	0	0	39		25	0	0	2	24	
AlexNet	H	38	36	3	0	0	94.16%	26	24	1	0	0	91.17%
	F1	39	2	34	1	0		25	2	20	0	0	
	F2	38	0	2	36	0		26	0	4	24	0	
	F3	39	0	0	1	39		25	0	0	2	25	
BPANN	H	38	36	2	0	0	94.16%	26	24	1	0	0	94.11%
	F1	39	1	35	3	0		25	2	23	0	0	
	F2	38	1	2	35	0		26	0	1	25	1	
	F3	39	0	0	0	39		25	0	0	1	24	

[a] NC: the number of samples in the calibration set.

[b] NP: the number of samples in the prediction set.

[c] CR: correct identification rate.

Table S3. Clssification performance of LDA, KNN, LeNet5, AlexNet and BPANN models for identification of sample.

Grade	Index	LDA		KNN		LeNet5		AlexNet		BPANN	
		Train	Test	Train	Test	Train	Test	Train	Test	Train	Test
H	Sensitivity	0.8947	0.8462	0.9474	0.9231	0.8947	0.8846	0.9474	0.9231	0.9474	0.9231
	Specificity	0.9138	0.9474	0.9741	0.9868	0.9828	0.9868	0.9741	0.9868	0.9828	0.9868
	Accuracy	0.9091	0.9216	0.9675	0.9706	0.9610	0.9608	0.9675	0.9706	0.9740	0.9706
	Error	0.0909	0.0784	0.0325	0.0294	0.0390	0.0392	0.0325	0.0294	0.0260	0.0294
F1	Sensitivity	0.5641	0.6400	0.8462	0.8000	0.8462	0.8000	0.8718	0.8000	0.8974	0.9200
	Specificity	0.9565	0.9610	0.9913	0.9351	0.9739	0.9610	0.9739	0.9740	0.9652	0.9740
	Accuracy	0.8571	0.8824	0.9545	0.9020	0.9416	0.9216	0.9481	0.9314	0.9481	0.9608
	Error	0.1429	0.1176	0.0455	0.0980	0.0584	0.0784	0.0519	0.0686	0.0519	0.0392
F2	Sensitivity	0.8947	0.8846	0.9474	0.8077	0.9737	0.9231	0.8718	0.9231	0.9211	0.9615
	Specificity	0.9224	0.9211	0.9569	0.9474	0.9483	0.9342	0.9828	0.9474	0.9828	0.9868
	Accuracy	0.9156	0.9118	0.9545	0.9118	0.9545	0.9314	0.9740	0.9412	0.9675	0.9804
	Error	0.0844	0.0882	0.0455	0.0882	0.0455	0.0686	0.0260	0.0588	0.0325	0.0196
F3	Sensitivity	1.0000	1.0000	1.0000	1.0000	1.0000	0.9600	1.0000	1.0000	1.0000	0.9600
	Specificity	0.9913	0.9610	0.9913	0.9740	1.0000	0.9740	0.9913	0.9740	1.0000	0.9870
	Accuracy	0.9935	0.9706	0.9935	0.9804	1.0000	0.9706	0.9935	0.9804	1.0000	0.9804
	Error	0.0065	0.0294	0.0065	0.0196	0.0000	0.0294	0.0065	0.0196	0.0000	0.0196

Experimental section

Multivariate analysis

PCA is a linear, unsupervised classification method. The purpose of this analysis involves approximating statistical variables with a limited number of possible linear combinations (so-called principal components) to structure, simplify and account for large data sets [1]. PCA is use linear transformation to map an n-dimensional feature to a k-dimensional space (k < n); the resulting k-dimension is a new orthogonal feature, and the k-dimensional feature is called the principal component (PC). PCi represents the ith principal component. The proportion of PCi variance to the total variance is called the contribution rate. A higher value of the contribution rate indicates a stronger ability to synthesize the information of the PCi. Analysis of the first ten principal component eigenvalues selected by PCA due to the cumulative contribution rate was 99.64% in this study (Table S1).

LDA is a technique commonly used for supervised pattern recognition. The ratio of maximizing inter-class distance to intra-class distance for maximum discrimination [2]. The PCA method was used in this study to reduce the dimensionality of the sensor data and use less variable information to represent the original sensor information. In this study, PCA and LDA were combined. The PCA method reduces the dimensionality of multidimensional variables and solves the problem of the small sample set of the LDA algorithm.

KNN identification model selects the closest points in the n-dimensional space with unknown samples in a range of K values [3]. In our study, a five-fold cross-validation method was applied to determine the optimal model. In this method, all 256 data pattern in each system has been divided into two groups, (a) calibration set which is used to train the classifier and (b) prediction set which is used to estimate the error rate of the trained classifier. The dataset has 64 samples in each of the five grades. For each of the five experiments, 80% of the total data (i.e. 51 out of 64 from each class/grade) are randomly selected and used to train the classifier. Rest 20% data are used for testing

purposes. The advantage of five-fold cross-validation is that all the feature sets are eventually used for both training and testing.

BPANN is a typical feed-forward multi-layer network composed of multiple layers of neurons (input layer, one or more hidden layers, and an output layer) connected unidirectionally from input to output (weights) [4]. In this study, the parameters are optimized through the model with the learning rate set to 0.1, the maximum number of training sessions set to 1000, the training accuracy set to 0.001, and the neurons all using sigmoid-type transfer functions. The optimal BPANN was evaluated by the minimal mean square error (MSE) value [5]. The BPANN optimization model was obtained analytically using different numbers of PCs (from 1 to 10) as neurons in the input layer.

To apply convolution to one-dimensional data, it is necessary to raise the dimensionality of the data, transforming it into a batchsize*features*1 format, and then performing convolution operations using convolution kernels. In the network design, this experiment used convolution kernels with a size of 3. Using excessively large convolution kernel sizes can lead to errors in small-dimensional inputs and inaccurate data fitting issues. The tanh activation function was employed to enhance the model's ability to fit nonlinear patterns, effectively achieving nonlinear fitting. we implemented the following designs to prevent data overfitting and information leakage: First, we used causal convolution to prevent the model from leaking future information and to address overfitting issues. Secondly, to further prevent data leakage, we normalized only the training set and did not normalize the validation set. Categorical Cross-Entropy function was utilized as the loss function for backpropagation to update model parameters, enhancing the model's fitting capability. Finally, the four output results from the last fully connected layer are obtained, and these four-dimensional outputs are processed through the softmax function to obtain the probabilities for each category.

LeNet is a classic deep convolutional neural network model, which is a typical feedforward multi-layer network consisting of multiple layers of neurons[6]. It includes the basic modules of deep learning convolutional neural networks: convolutional layers, pooling layers, and fully connected

layers. In the LeNet5 network model constructed in this study, there are a total of 7 layers (the input layer is not considered part of the network structure), consisting of 2 convolutional layers, 2 pooling layers, and 3 fully connected layers. The model obtains four outputs through the last fully connected layer, and these outputs, with four dimensions, are then passed through the softmax function to obtain the probability of each category.

AlexNet has a deeper network structure and introduces the LRN layer, which creates a competition mechanism for the activity of local neurons. This mechanism makes the relatively larger values more significant and suppresses neurons with smaller feedback, thereby enhancing the model's generalization ability[7]. During training, Dropout is employed to randomly ignore a portion of neurons to prevent overfitting. The AlexNet network model constructed in this study comprises a total of 11 layers, consisting of 2 convolutional layers, 2 pooling layers, 2 normalization layers, 1 flattening layer, 1 Dropout layer, and 3 fully connected layers. The data is passed through forward propagation, and finally, the softmax function is used to convert the output into a probability distribution, obtaining the probability for each category.

[1] A. Winkler, M. Rauwolf, J.H. Sterba, P. Wobrauschek, C. Streli, A. Turyanskaya, Total reflection X-ray fluorescence analysis of elemental composition of herbal infusions and teas, J Sci Food Agric, 100(2020) 4226-36.

[2] M. Esteki, N. Memarbashi, J. Simal-Gandara, Classification and authentication of tea according to their geographical origin based on FT-IR fingerprinting using pattern recognition methods, Journal of Food Composition and Analysis, 106(2022) 8.

[3] Y.K. Kwon, Y.S. Bong, K.S. Lee, G.S. Hwang, An integrated analysis for determining the geographical origin of medicinal herbs using ICP-AES/ICP-MS and H-1 NMR analysis, Food Chemistry, 161(2014) 168-75.

[4] Q.S. Chen, J.W. Zhao, S. Vittayapadung, Identification of the green tea grade level using electronic tongue and pattern recognition, Food Res Int, 41(2008) 500-4.

[5] C.A. Emilio, J.F. Magallanes, M.I. Litter, Chemometric study on the TiO2-photocatalytic degradation of nitrilotriacetic acid, Anal Chim Acta, 595(2007) 89-97.

[6] Y. Sun, S. Liu, T. Zhao, Z. Zou, B. Shen, Y. Yu, et al., A new hydrogen sensor fault diagnosis method based on transfer learning with LeNet-5, Frontiers in Neurorobotics, 15(2021) 664135.

[7] G. Mingang, G. He, Research on AlexNet Improvement and Optimization Method, Journal of Computer Engineering & Applications, 56(2020).
